# Binding studies of promethazine and its metabolites with human serum albumin by high-performance affinity chromatography and molecular docking in the presence of codeine

**DOI:** 10.1007/s00216-024-05409-3

**Published:** 2024-07-04

**Authors:** Maria Miguel Coelho, Rita Lima, Ana Sofia Almeida, Pedro Alexandrino Fernandes, Fernando Remião, Carla Fernandes, Maria Elizabeth Tiritan

**Affiliations:** 1 Laboratory of Organic and Pharmaceutical Chemistry, Department of Chemical Sciences, Faculty of Pharmacy of the University of Porto, 4050-313 Porto, Portugal; 2 CIIMAR-Interdisciplinary Center for Marine and Environmental Research University of Porto, Porto de Leixões Cruise Terminal, 4450-208 Matosinhos, Portugal; 3 UCIBIO-Applied Molecular Biosciences Unit, Laboratory of Toxicology, Department of Biological Sciences, Faculty of Pharmacy, University of Porto, 4050-313 Porto, Portugal; 4 LAQV, REQUIMTE, Departamento de Química E Bioquímica, Faculdade de Ciências, Universidade Do Porto, Rua Do Campo Alegre, S/N, 4169-007 Porto, Portugal; 5 1H-TOXRUN – One Health Toxicology Research Unit, University Institute of Health Sciences (IUCS), CESPU, CRL, 4585-116 Gandra, Portugal

**Keywords:** “Purple Drank”, Promethazine, Human serum albumin, Binding affinity, High-performance affinity chromatography, Docking

## Abstract

“Purple Drank”, a soft drink containing promethazine (PMZ) and codeine (COD), has gained global popularity for its hallucinogenic effects. Consuming large amounts of this combination can lead to potentially fatal events. The binding of these drugs to plasma proteins can exacerbate the issue by increasing the risk of drug interactions, side effects, and/or toxicity. Herein, the binding affinity to human serum albumin (HSA) of PMZ and its primary metabolites [*N*-desmethyl promethazine (DMPMZ) and promethazine sulphoxide (PMZSO)], along with COD, was investigated by high-performance affinity chromatography (HPAC) though zonal approach. PMZ and its metabolites exhibited a notable binding affinity for HSA (%*b* values higher than 80%), while COD exhibited a %*b* value of 65%. To discern the specific sites of HSA to which these compounds were bound, displacement experiments were performed using warfarin and (*S*)-ibuprofen as probes for sites I and II, respectively, which revealed that all analytes were bound to both sites. Molecular docking studies corroborated the experimental results, reinforcing the insights gained from the empirical data. The in silico data also suggested that competition between PMZ and its metabolites with COD can occur in both sites of HSA, but mainly in site II. As the target compounds are chiral, the enantioselectivity for HSA binding was also explored, showing that the binding for these compounds was not enantioselective.

**Supplementary Information:**

The online version contains supplementary material available at 10.1007/s00216-024-05409-3.

## Introduction

**Fig. 1:**
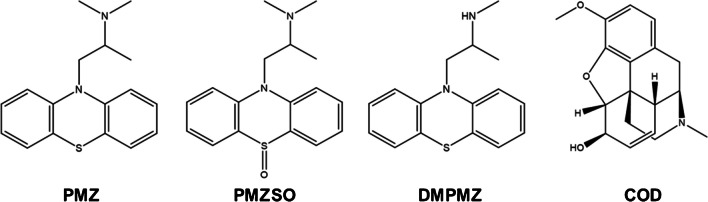
Chemical structures of promethazine (PMZ), promethazine sulphoxide (PMZSO), *N*-desmonomethyl promethazine (DMPMZ), and codeine (COD)

Promethazine (PMZ, Fig. 1) is one of the most important compounds among the phenothiazine derivatives; however, it is structurally different from the neuroleptic phenothiazines, acting mainly as a strong H1 receptor antagonist (antihistamine) [1, 2]. Additionally, PMZ has high anticholinergic properties, since it blocks acetylcholine responses through the mediation of muscarinic receptors [3]. This blockade of acetylcholine responses explains why an overdose of PMZ can lead to anticholinergic toxidrome, where signs and symptoms such as dry mouth, difficulty swallowing, mydriasis with blurred vision, and photophobia can be seen. Moreover, at a cardiovascular level, sinus tachycardia can occur, and blood pressure can drop due to peripheral vasodilation or rise due to agitation [3]. It is known that PMZ is rapidly metabolized into its main metabolites: promethazine sulphoxide (PMZSO) and *N*-desmonomethyl promethazine (DMPMZ) (Fig. 1). Studies of both PMZ and metabolites are scarce but are pivotal for a better understanding of the pharmacodynamics, pharmacokinetics, and toxicological effects of PMZ [4]. DMPMZ demonstrated to be even more cytotoxic than PMZ towards differentiated SH-SY5Y cells, highlighting the importance to study the metabolites [5].

Recently, a great concern regarding PMZ is its combination with codeine (COD) and alcohol in a soft drink called “Purple Drank” that has become a worldwide phenomenon due to its hallucinogenic effects [6, 7]. COD is an opioid analgesic drug mainly used to treat pain, coughing, and diarrhoea, but is also commonly consumed as a recreational drug [8]. The combination of these two drugs and its consumption in large quantities can be a serious problem because both drugs act at the central nervous system leading to a prolongation and intensification of the sedative effects of each of them. Its drug association is responsible for an increase in potentially fatal events, including delirium, respiratory depression, overdose, and neuroleptic malignant syndrome [3, 9]. It is also important to highlight that drug interactions involving opioid analgesics, such as COD, can be very problematic, as opioids have a narrow therapeutic index, which can be associated with serious toxicity [10].

Another noteworthy aspect that is often associated with drug interactions and, consequently, toxicity is related to the binding of drugs to plasma proteins [11]. The co-administration of two drugs may alter their affinity to plasma proteins as competitive binding occurs (or even can saturate albumin), thus influencing pharmacokinetic interactions, which result in a change of the concentration of the biologically active fraction of one or two drugs, subsequently influencing the therapeutic effect, side effects, and/or toxicity [12].

There are numerous reports recognizing that the competition between two drugs for binding to plasma proteins can significantly impact their pharmacological and/or toxicological profile [13]. Warfarin (anticoagulant) is a classic example of a drug that has a high affinity for human serum albumin (HSA), the most abundant plasma protein, exhibiting drug interactions and, therefore, toxicity when other drug with HSA affinity is co-administered [13, 14]. For instance, when warfarin is co-administered with phenylbutazone (anti-inflammatory, antipyretic, and analgesic) or sulfamethoxazole (antibiotic) or clofibrate (reduces lipid level in blood), there is an elevated risk of bleeding in patients. This is attributed to the competition between both drugs for the same HSA site, and, consequently, there is a greater amount of unbound warfarin (free and active form), leading to an increased risk of bleeding [14]. Saturation of HSA may also occur when the blood concentration of drugs is high due, for example, to an excessive and uncontrolled drug consumption, resulting in an increase in their free fraction. This phenomenon is important especially for drugs that have high bind affinity to HSA, where small variations in the amount of free fraction may have a consequent impact on the therapeutic and/or toxicity effects of the drugs [15].

Monitoring the reversible binding of drugs to HSA is essential to understand how drugs behave in the body as it controls the free and active concentration of a drug which affects its distribution, metabolism, and excretion, determining the overall pharmacological/toxicological effect [15–17]. Most of the studies consider that the volume of distribution and the concentration of a drug at the target site is interconnected with the quantity of the unbound fraction of the drug [18, 19].

Several methods are used to study drug affinity with serum proteins [20–22]. Among them, high-performance affinity chromatography (HPAC) proved to be a very useful and efficient technique to study intermolecular interactions between HSA and drugs [23]. HPAC is advantageous as it features high precision and speed, easy handling, high reproducibility and applicability, and possibility of working in near-physiological conditions, among others [19]. Displacement studies can also be carried out using HPAC to understand which specific sites the drugs bind to HSA [24, 25]. Another advantage is the enantioseparation of chiral drugs and analytes [26–28] as the interactions with HSA may be enantioselective [13].

Another convenient approach to analyse HSA binding sites and shed light on the recognition mechanisms is the in silico method, such as molecular docking approach [29]. Docking studies can be used to model the interaction between a drug and a protein, such as HSA, at the atomic level, which allows to characterize the behaviour of the drug in the target protein binding site [30].

Information in literature regarding the binding of PMZ and COD to HSA is scarce [31–33]. Moreover, to the best of our knowledge, no reports on the interaction between HSA and PMZ metabolites were found. In addition, only one study concerning the decrease of PMZ binding affinity to HSA in the presence of flavonoids was reported [31].

This work aims to understand how PMZ and its main metabolites (PMZSO, DMPMZ), and COD interact and bind to HSA individually. Subsequently, it was important to verify whether COD may compete with PMZ and its metabolites in the binding to the protein. HPAC, using an HSA column, and molecular modelling docking were the tools that allowed a rapid and effective analysis of drug–protein binding.

The main novelty of this work was to analyse for the first time by HPAC and docking methods the binding affinity of PMZ, its main metabolites and COD to HSA, to understand the recognition mechanisms and identify the sites as well as the interactions between these compounds and the protein. Another novelty was to infer that competition between PMZ and its metabolites with COD may occur when both drugs are co-administrated.

## Experimental

### Chemicals

The PMZ (98.1% pure) was acquired from Dr. Ehrenstorfer GmbH (LGC, Middlesex, UK), DMPMZ (98.7% pure) from TRC (Toronto, Canada), PMZSO (99.0% pure) from Merck (Darmstadt, Germany), warfarin (98% pure), and (*S*)-ibuprofen (99% pure) from Sigma-Aldrich Co. (St. Louis, Missouri, USA). Potassium phosphate and phosphoric acid were of analytical grade and obtained from Sigma-Aldrich Co. (St. Louis, Missouri, USA) or Carlo Erba (Barcelona, Spain). Ethanol (EtOH), n-hexane (Hex), 2-propanol (2-PrOH), and acetonitrile (ACN), for HPLC, were purchased from Sigma-Aldrich Co. (St. Louis, Missouri, USA). Ultrapure water (UPW) was produced by a Millipore Milli-Q system (conductivity ≤ 0.1 µS cm^−1^) (Millipore, Bedford, MA, USA).

### Instrumental and chromatographic conditions

The UHPLC system used in the chromatographic analyses was a Dionex UlltiMate 3000 system (Thermo Fisher Scientific Inc., Waltham, MA, USA), equipped with a 3000 quaternary pump, a 3000 autosampler, and a 3000 variable wavelength detector. The chromatographic data was analysed with Chromeleon™ software version 7.2 Ultimate (Thermo Fisher Scientific Inc., Waltham, MA, USA). A Chiralpak® HSA column (150 × 40 mm I.D., 5 µm particle size) from Chiral Technologies Europe (Daicel Chemical Industries, Ltd., Osaka, Japan) was used to perform both HSA binding affinity and displacement studies. The flow rate used was 0.5 mL min^−1^, and the chromatograms were monitored by UV detection at 254 nm. The sample injections (10 µL) were carried out in triplicate, and the analysis temperature was 25 ± 2 °C. The analyses were performed in reversed-phase elution mode using potassium phosphate buffer solution (67 mM, pH 7.0) for binding affinity experiments. To verify whether there was enantioselectivity, other mobile phases were explored, namely mixtures of potassium phosphate buffer (10 mM; pH 7.0) with different percentages of ACN or 2-PrOH as organic modifiers. The mobile phases were prepared in a volume/volume ratio (*v*/*v*), filtered through polyamide membrane filters of 0.2 µm pore size from Whatman® GmbH (Dassel, Germany) and degassed in an ultrasonic bath (Sonorex Digitec, Bandelin) for 20 min prior to use. Before the analyses, all mobile phases were left overnight conditioning to guarantee a thorough stabilization for reproducibility of results. Competition studies using zonal elution were conducted by employing warfarin and (*S*)-ibuprofen as site-specific probes for Sudlow site I and Sudlow site II, respectively [34]. Data were analysed using GraphPad Prism 9.3 (GraphPad Software Inc.).

### Samples and buffer preparation

For HSA binding affinity and displacement studies, stock solutions of all analytes (PMZ, PMZSO, DMPMZ, and COD) were prepared in EtOH at the concentration of 1 mg mL^−1^ and further diluted with the mobile phase to a final concentration of 50 μg mL^−1^. The probe solutions of warfarin and (*S*)-ibuprofen, used in displacement experiments, were prepared in the mobile phase, i.e., a mixture of potassium phosphate buffer (67 mM, pH 7.0): ACN (99:1 *v*/*v*). All solutions were stored at 4 °C. Probe solutions were added to the mobile phase at six concentrations ranging from 5 to 30 µM (5, 10, 15, 20, 25, and 30 µM). These concentrations were selected based on previous displacement studies [24]. All working solutions were filtered through polyamide membrane filters of 0.2 µm pore size from Whatman® GmbH (Dassel, Germany). Aqueous buffer of potassium phosphate was prepared by adjusting the pH of 67 mM solution of K_2_HPO_4_ to 7.0 with a solution of saturated NaOH. The aqueous buffer pH was controlled by a Gondo® PL-700PV pH monitor.

### Bound fraction determination

Binding affinity studies using HPAC can be performed by two main approaches: frontal or zonal elution [35]. In frontal analysis, the analyte is continuously injected into a column that contains the immobilized protein. As the analyte binds to the immobilized protein, the column will eventually saturate increasing the amount of analyte that elutes from the column resulting in the formation of a breakthrough curve. In zonal analysis, the approach selected for this work, a small amount of analyte is injected into a column, while a mobile phase of known composition passes through at a steady flow rate. In this approach, the retention of the injected analyte is directly related to the analyte’s interaction with the immobilized ligand in the column and can be described with Eq. (1) [35, 36].

1 $$k= \frac{({K}_{A1}{n}_{1}+.. .. .. + {K}_{An}{n}_{n}){m}_{L}}{{V}_{M}}$$

where the retention factor (*k*) of the analyte is associated with the number of binding sites and the corresponding equilibrium constants at each site (*K*_A_). *m*_L_ represents the total moles of binding sites in the column and *V*_M_ the void volume. In this work, the retention factor was calculated through Eq. (2) [37]:

2 $$k=\frac{{t}_{r}-{t}_{0}}{{t}_{0}}$$

wherein *t*_r_ is the retention time of the solute and *t*_0_ is the dead time that was considered to be equal to the peak of the solvent front and was taken from each particular run.

One of the main applications of the zonal elution approach is to determine the binding affinity between an analyte and the immobilized ligand. The bound fraction can be associated with the retention factor through Eq. (3):

3 $$k= \frac{b}{f}$$

where *b* represents the ligand bound fraction of the analyte and *f* the free fraction of analyte in solution. Since the sum of the bound and free fractions should be equal to 1, by rearranging Eq. (3), *b* can be calculated using only the retention factor. In this work, the %*b* was calculated in only aqueous phase (analysed in triplicate), according to Eq. (4) [24, 37]:

4 $$\%b=\frac{\text{k}}{1+\text{k}}\times 100$$

### Displacement experiments

The experimental technique employed to perform displacement studies and identify the binding site(s) of analytes using the zonal elution approach involved the following steps [38]: (1) injecting the drug, “solute”, into the column containing the immobilized protein and measuring its retention factor, *k*; (2) introducing the other drug, the competitor, into the mobile phase and systematically increasing its concentration in a series of experiments; (3) assessing the impact of “competitor” concentrations on the *k* values of the solute [13]. Warfarin and (*S*)-ibuprofen were the competitor used to perform these experiments [34].

The plot of 1/*k* versus the concentration of the competing agent can be used to verify the effect of the competing agent (I) on the retention of each analyte (A), being represented by the following equation [37]:

5 $$\frac{1}{{k}_{A}}=\frac{{V}_{M}{K}_{I}[I]}{{K}_{A}{m}_{L}}+\frac{{V}_{M}}{{K}_{A}{m}_{L}}$$

where *k*_A_ is the measured retention factor of the analyte A, *V*_M_ is the void volume of the column, *K*_I_ is the equilibrium association constant of the displacer I for the single binding site of A, [*I*] is the molar concentration of the competitor in the mobile phase, *K*_A_ is the equilibrium association constant of A for its binding site, and *m*_L_ is the total moles of binding sites of A in the column [37].

In this case, if there is a direct competition at a single site between the analyte and the competing agent, a straight line with a positive slope is observed on the reciprocal retention factor (1/*k*) against the [*I*] graph, whereas a non-competitive relationship lacks of correlation [24, 37]. If competition is observed, the equilibrium association constant (*K*_I_) of the competitor at the analyte binding site can be obtained using the ratio between the slop and intercept by the following equation [24]:

6 $$\frac{Slope}{Intercept}=\frac{\left(\frac{{V}_{M}{K}_{I}}{{K}_{A}{m}_{L}}\right)}{\left(\frac{{V}_{M}}{{K}_{A}{m}_{L}}\right)}={K}_{I}=\frac{1}{{K}_{D}}$$

### Computational

The X-ray crystal structure of HSA was downloaded from the Protein Data Bank (PDB code: 2BXG) [39]. The structures of all analytes (PMZ, DMPMZ, PMZSO, and COD) were modelled in GaussView 5.0 (Gaussian, Inc., 340 Quinnipiac St Bldg 40 Wallingfor, CT, USA) and Open Babel 3.0.0 [40, 41]. The docking calculations were done considering only the structural features (binding pockets) assumed to be essential for the interactions between HSA and analytes. AutoDock Vina 4 (Molecular Graphics Lab, CCSB, The Scripps Research Institute) was used for docking calculations between HSA and the analytes [42]. For the docking studies, a rigid HSA receptor was considered, and the ligands (analytes) were considered as flexible.

AutoDock Vina was run using an exhaustiveness of 8 and two grid boxes according to the interaction sites, namely sites I and II of HSA. The graphical interface used to build the grid boxes was the AutoDock/Vina plugin from Pymol. Each box was centred in the centre of mass of the selection encompassing the residues comprising each HSA site. For site I, the grid box was centred in X: 5.00; Y: − 8.00; Z: 7.00 and had the dimensions (Å) of X: 20.6, Y: 17.6, and Z: 18.0, and for site II, the grid box was centred in X: 6.00; Y: 2.00; Z: − 14.00 and had the dimensions (Å) of X: 20.6, Y: 16.9, and Z: 16.5. The lowest binding affinity conformations were investigated, in a total of up to nine different conformations. PyMOL version 2.3.0 (Schrödinger, New York, NY, USA) was used for visual inspection of results and graphical representations [43].

Lipophilicity was measured by calculating the partition coefficient between two solvents, n-octanol and water (logP_o/w_) using SwissADME software [44].

## Results and discussion

### Determination of HSA binding affinity

HPAC is the most widespread and broadly accepted methodology for exploring drug–protein interactions [23]. Within HPAC framework, analytes are introduced into the column, enabling an exploration of their interaction with the immobilized affinity protein. This assessment reveals that compounds with a heightened affinity for the protein experience delayed elution compared to those with lower affinity or no affinity at all [19].

To evaluate the binding affinity of PMZ, their metabolites (DMPMZ and PMZSO) and COD (Fig. 1) to HSA, a Chiralpak® HSA column was chosen with a zonal chromatography approach [24]. A solution of potassium phosphate buffer (pH 7.0; 67 mM) was used as mobile phase. This buffer was selected due to the stability of HSA in phosphate buffers [45]. In addition, this type of buffer at 67 mM proved to mimic more closely the physiological conditions of human body [18, 46, 47]. In fact, most of the reported studies used potassium phosphate buffer at 67 mM for HSA binding affinity evaluation by HPAC [20]. Considering that all analytes showed satisfactory elution without the need of organic modifiers in the mobile phase, a solution of 100% buffer (pH 7.0; 67 mM) was applied as mobile phase to assess the binding affinity of the compounds to HSA.

The bound fraction (%*b*) to HSA was subsequently calculated according to Eq. (4), using the *K* values determined from Eq. (2) (Table 1). As controls, the following three compounds were used: chlorpromazine, indomethacin, and metronidazole, whose %*b* values to HSA are already described [48–50]. For calculation of experimental %*b* of the controls, it was necessary to added different proportions of organic modifier (ACN) to the mobile phase ranging from 2 to 20%. Then, to calculate the %*b* values in 100% buffer, an extrapolation was performed by linearly plotting the log *k* values against the percentage of organic modifier in the mobile phase [28]. It was found that the experimental %*b* values were similar to those described in the literature (Table 1).

**Table 1:** Bound fraction (%*b*) to HSA and theoretical partition coefficient (logP_o/w_) values of PMZ, its metabolites (PMZSO and PMZND), COD and controls

Analyte	Retention time (min)	*k*	%*b*	log P_O/W_^[a]^
PMZ	62.01	40.11	98	3.75
PMZSO	14.14	3.99	80	2.98
DMPMZ	49.12	16.34	94	3.57
COD	7.49	1.89	65	1.75
Chlorpromazine	-	-	98.1 (> 90)[48]	-
Indomethacin	-	-	96.9 (97.8)[49]	-
Metronidazole	-	-	19.8 (20.0)[50]	-

^[a]^Values obtained from the SwissADME web tool provided by SIB (Swiss Institute of Bioinformatics)

As shown in Table 1, for the studied analytes, the %*b* values ranged from 65 to 98%. Contrasting to COD, PMZ and its metabolites (DMPMZ and PMZSO) exhibited a notable binding affinity for HSA, as their %*b* values surpass the threshold of 80% [24, 49]. Moreover, PMZ and DMPMZ displayed the highest affinity for HSA being the more retained compounds on HSA column, with %*b* values of 98 and 94%, respectively. This high affinity was predictable because HSA has an increase affinity for low molecular weight, lipophilic, and negatively charged compounds [28, 49, 51]. Although PMZ and its metabolites are not negatively charged, they are lipophilic molecules as evidenced by their partition coefficient in n-octanol/water (logP_o/w_) values (Table 1).

Lipophilicity was assessed by determining the logP_o/w_ utilizing the SwissADME web tool. This software offers free access to a suite of rapid yet reliable predictive models for physicochemical and pharmacokinetic properties [44]. The high affinity of PMZ and DMPMZ for HSA establishes a robust connection with lipophilicity, given that they are the most lipophilic compounds, with logP_o/w_ values of 3.75 and 3.57, respectively. Conversely, in the case of COD, it has the lowest logP_o/w_ value, 1.75, which also aligns appropriately, consistent with COD being classified as a moderately lipophilic compound [52].

Additionally, it was possible to identify a relationship between the chemical structures of PMZ and its metabolites with their lipophilicity profile and, consequently, its affinity to HSA. The DMPMZ metabolite, obtained from *N*-dealkylation of PMZ [53], only has one methyl group (nonpolar group) on the amine of the side chain of the central ring of the phenothiazine scaffold, unlike PMZ that has two methyl groups. This structural difference makes DMPMZ less lipophilic and with lower affinity for HSA. Regarding PMZSO metabolite, it resulted from hepatic oxidation of the sulphur in the central ring of the tricyclic system to afford a sulphoxide [54, 55], a more polar group. The presence of this polar group led to a decrease in lipophilicity and, therefore, lower affinity for HSA.

The high binding affinity of PMZ for HSA was previously demonstrated by Zhao et al*.* [56], though HPAC using a column containing immobilized HSA and applying a novel mathematical model. The association constant of PMZ binding to HSA was determined to be 1.24 ± 0.14 × 10^4^ M^−1^ [56]. A similar association constant (1.57 ± 0.20 × 10^4^ M^−1^) was reported by He et al*.* [31], using fluorescence and adsorption spectroscopy. Moreover, in another study using the same spectroscopic technology, the binding affinity of PMZ to bovine serum albumin was explored, gave a binding constant of 1.40 ± 0.04 × 10^4^ M^−1^ [57]. Interestingly, these data, which were obtained from different analytical methods, agree well with the results obtained in this work, as all demonstrated that PMZ has a high binding affinity for HSA. To the best of our knowledge, there are no reports on the interaction between HSA and PMZ metabolites (DMPMZ and PMZSO). Regarding COD, in a study involving equilibrium and dynamic dialysis, very low binding percentage (about 29%) was achieved for COD to HSA [32].

As PMZ and its metabolites are chiral and tested as racemates, the enantioselectivity on HSA was also explored though additional experiments, carried out using other mobile phases, namely mixtures of potassium phosphate buffer (10 mM; pH 7.0) with different percentages of ACN or 2-PrOH as organic modifiers (ranging from 0 to 15%). ACN and 2-PrOH were selected since they are the most used organic modifiers for this type of studies on HPLC HSA columns [20]. To avoid the protein denaturation and, consequently, shortening the column lifetime, a low percentage of organic modifier is mandatory for this type of columns. Additionally, the presence of organic modifier may interfere with the reversible spatial conformation of the protein being responsible for modification in the number of available binding sites [47, 58]. It was found that, under the studied chromatographic conditions, no enantioseparation was observed. However, evidence of the enantioselective binding of PMZ to human plasma proteins was demonstrated by the incubation of samples containing plasma and the racemate followed by ultrafiltration of the mixture and enantioseparation using affinity electrokinetic chromatography-partial filling technique and HSA as chiral selector [59] and capillary zone electrophoresis [60]. To the best of our knowledge, the enantioseparation of PMZ using a HPLC column containing immobilized HSA was not investigated before.

### Displacement studies

HSA is a 66.5-kDa globular protein with around 585 amino acid residues containing a sequence of 17 tyrosines (Tyr), 6 methionines (Met), 1 tryptophan (Trp-214), 17 disulphide bridges, and a free thiol group that maintains a characteristic heart-shaped conformation [61]. The crystallographic structure of HSA reveals three homologous α-helical domains (I–III). Each domain consists of ten helices, further divided into two subdomains, A and B, which contain six and four α-helices, respectively. The main sites where most drugs and other compounds (endogenous and exogenous) bind to HSA are primarily located in the hydrophobic cavities of subdomain IIA (site I) and subdomain IIIA (site II) [62]. Both site I and site II are well characterized and provide a versatile environment capable of accommodating a large number of diverse array of compounds [34].

In a previous study using fluorescence spectroscopy, it was found that the binding site of PMZ on HSA was located in site I (subdomain IIA) [57], but the binding sites of PMZ metabolites were not explored. To enhance understanding of the binding sites of PMZ and identify the binding sites of the metabolites of PMZ (DMPMZ and PMZSO) on HSA, displacement experiments by zonal chromatography were carried out. Another key objective was to analyse the possibility of binding competition between these target compounds (PMZ, DMPMZ, and PMZSO) and COD for HSA.

When two drugs (drugs 1 and 2) simultaneously interact with the HSA immobilized in the column, competitive or non-competitive binding may occur [31]:

1. Competitive binding
  $$\text{HSA}\stackrel{+ {\text{drug1}}}{\to }\text{HSA}-\text{drug }1 \stackrel{+ {\text{drug2}}}{\to }\text{ HSA}-\text{drug}2 +\text{ drug}1$$
2. Non-competitive binding
  $$\text{HSA}\stackrel{+ {\text{drug1}}}{\to }\text{HSA}-\text{drug }1 \stackrel{+ {\text{drug2}}}{\to }\text{ drug}1-\text{HSA}-\text{drug}2$$

**Fig. 2:**
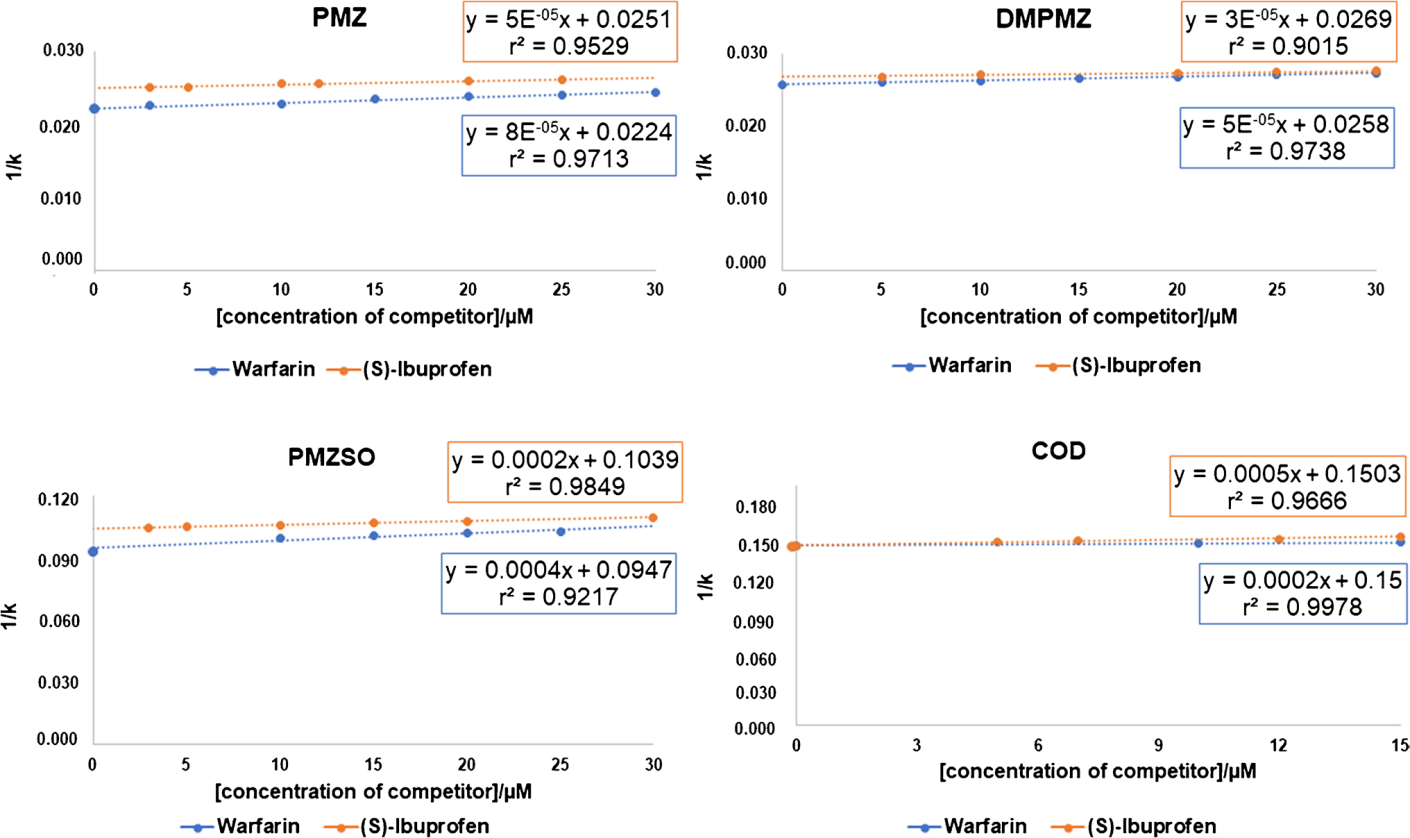
Displacement chromatography experiments with PMZ, its metabolites, and COD in the absence and presence of increasing concentrations of the competitors, warfarin and (*S*)-ibuprofen, expressed by the plot of 1/*k* of the analyte versus the concentrations of the competitor compounds

In this study, the selected competitors were warfarin and (*S*)-ibuprofen, known to bind to specific binding sites on HSA, namely site I and site II, respectively. Various concentrations of the competitors were introduced into the mobile phase, according to previous reported studies [13, 24]. The displacements of the analytes in the presence of increasing concentrations of competitors are shown in Fig. 2. The chromatographic data was plotted as 1/*k* versus the concentration of the competitor.

As shown in Fig. 2, for PMZ a shortening of the *k* values was observed with the increase of the concentration of warfarin, being the decrease statistically significant (*P* < 0.05, Figure S1, SI). Moreover, the plot of 1/*k* versus competitor concentration revealed a linear relationship with a positive slope. This result confirms that PMZ competes with warfarin, agreeing with the previously reported data obtained by using fluorescence spectroscopy, which indicated that PMZ binds to HSA site I [31]. Nevertheless, similar behaviour was observed when (*S*)-ibuprofen was used as the competitor; thus, indicating that PMZ also compete with (*S*)-ibuprofen, in site II.

For PMZ metabolites (DMPMZ and PMZSO) a decrease of the *k* values was also found upon increasing the concentration of both competitors, being the decrease statistically significant (*P* < 0.05). Similarly, a linear relation with a positive slope for the plot of 1/*k* versus competitor concentration was achieved for both analytes. These results indicate that DMPMZ and PMZSO also compete with warfarin and (*S*)-ibuprofen. Therefore, it can be hypothesized that both sites I and II are primarily involved in the binding of all these compounds to HSA. For PMZ, the straight line obtained by plotting 1/*k* versus the concentration of the warfarin revealed a higher slope indicating that PMZ exhibits more substantial competition with warfarin compared to (*S*)-ibuprofen.

Note that, in the case of COD, competition was also observed for both sites I and II. It was found that the increase of concentration of warfarin significantly changed the retention of COD (*P* < 0.05). The use of (*S*)-ibuprofen as the competitor (until 15 µM) resulted in a decrease of COD retention on the HSA column, being statistically significant (*P* < 0.05). From the concentration of 15 µM of (*S*)-ibuprofen (20, 25, and 30 µM), no significant changes were found for the k values (not shown) when compared to the values obtained for 15 µM which might suggest that saturation of site II on HSA occurred.

Once HSA binding sites have been identified for PMZ and its metabolites, and since COD also binds to site II, there is the possibility of COD competition with each of these compounds.

Meanwhile, the *K*_I_ values of the competitors were determined using Eq. 6, based on its competition effect [24]. The calculated values are collected in Table 2. Then, the *K*_I_ values were compared with the literature-reported affinity constants for warfarin and (*S*)-ibuprofen, which are 3.3 × 10^5^ M^−1^ and 2.7 × 10^6^ M^−1^, respectively [49]. This comparative analysis is very effective to predict the nature of competition between the analytes and the competitor, as well as the type of binding mechanism at the site [63]. If *K*_I_ values are of the same order of magnitude of the affinity constant found in literature for the competitors, direct competition should be considered between the analytes and competitors [63].

**Table 2:** *K*_I_ values of competitors, warfarin and (*S*)-ibuprofen, obtained by displacement chromatography analysis

Compound	K_I_ warfarin (M^−1^)	K_I_ (*S*)-ibuprofen (M^−1^)
PMZ	3.6 × 10^3^	2.0 × 10^3^
DMPMZ	2.3 × 10^3^	1.1 × 10^3^
PMZSO	4.2 × 10^3^	1.9 × 10^3^
COD	1.3 × 10^3^	3.3 × 10^3^

Herein, for both the competitors, the calculated *K*_I_ values were always much lower than that expected in case of a direct competition, thus suggesting an allosteric competition between the analytes and competitors with a non-cooperative binding mechanism [63].

In addition, other displacement experiments by zonal approach were performed aiming to confirm the multisite direct competition between PMZ and its metabolites with COD on HSA. Various solutions of COD were added to the mobile phase with concentrations ranging from 3 to 10 µM. Concentrations of COD up to 10 µM were not possible to introduce in the mobile phase as an increase in pressure in the column was found. Under these conditions, no significant differences were observed in the *k* values of PMZ and its metabolites versus the increase of COD concentration. One reason that can justify these results is the low concentrations of COD tested, which were not adequate to carry out a displacement study.

### Docking studies

Molecular docking is a very effective method for the characterization of recognition mechanisms at the molecular level and the understanding of the interactions between target analytes and HSA [64]. The results obtained from the displacement experiments revealed that PMZ, its metabolites, and COD were bound to both sites I and II of HSA. Moreover, no enantioselectivity was observed for PMZ and its metabolites on HSA.

Herein, molecular modelling and docking calculations were performed to achieve four main objectives: (a) to further confirm the binding sites of PMZ, its metabolites (DMPMZ and PMZSO), and COD on HSA; (b) to understand the recognition mechanisms behind the experimental results and identify the interactions between analytes and HSA; (c) to confirm the absence of enantioselectivity; and (d) to visualize whether PMZ and its metabolites could bind to site I and site II of the HSA: COD complex.

The docking scores of PMZ and its metabolites are presented in Table 3. Lower docking score means that the protein:analyte complex is more stable [65]. In the case of Autodock Vina, the score corresponds to the estimated binding free energy.

**Table 3:** Binding free energy for the top conformation for each enantiomer of the PMZ, its metabolites (DMPMZ and PMZSO), and COD. Binding free energies are shown in kcal/mol

Analytes	Binding free energy (site I)	Binding free energy (site II)
(*R*)-PMZ	− 7.2	− 7.2
(*S*)-PMZ	− 7.3	− 6.4
(*R*)-DMPMZ	− 7.2	− 4.3
(*S*)-DMPMZ	− 6.8	− 6.1
(*R*)-PMZSO	− 8.3	− 3.2
(*S*)-PMZSO	− 7.0	− 3.2
COD	− 7.8	− 6.2
Warfarin	− 9.8	−
(*S*)-Ibuprofen	−	− 7.7

As shown in Table 3, for the analytes that are experimentally described to have high affinity towards site I and site II of HSA were obtained binding free energies of − 9.8 kcal/mol (warfarin) and − 7.7 kcal/mol ((*S*)-Ibuprofen).

The overall results indicate that all analytes possess affinity for both sites (binding free energy range between − 3.2 and − 8.3 kcal/mol), although lower affinities are frequently observed at site I. In particular, PMZ metabolites could bind significantly better at site I, as the difference in binding free energy can range beyond 3 kcal/mol. These results suggest that the PMZ and COD might compete with both warfarin and (*S*)-ibuprofen, whereas PMZ metabolites (DMPMZ and PMZSO) should be more likely to compete with warfarin than (*S*)-ibuprofen. Therefore, there is an 83% agreement between docking free energies and experimental HPAC displacement data concerning the HSA binding site, representing a very good agreement between the experimental results and the in silico values [66].

The free energy difference between the enantiomeric pairs for most of the analytes in both HSA sites is less than 1 kcal/mol [67], except for DMPMZ at site II (for which binding free energies are also lower). These results support that, within the reported accuracy of docking calculations with AutoDock Vina (3 kcal/mol) [42], there should be no significant enantiodiscrimination, in line with experimental results.

A visual inspection of the binding conformations at each site of HSA was performed for all the analytes to interpret the binding free energies. Sites I and II each feature a pocket primarily composed of hydrophobic and positively charged residues, providing a versatile environment capable of accommodating a diverse array of compounds [13].

**Fig. 3:**
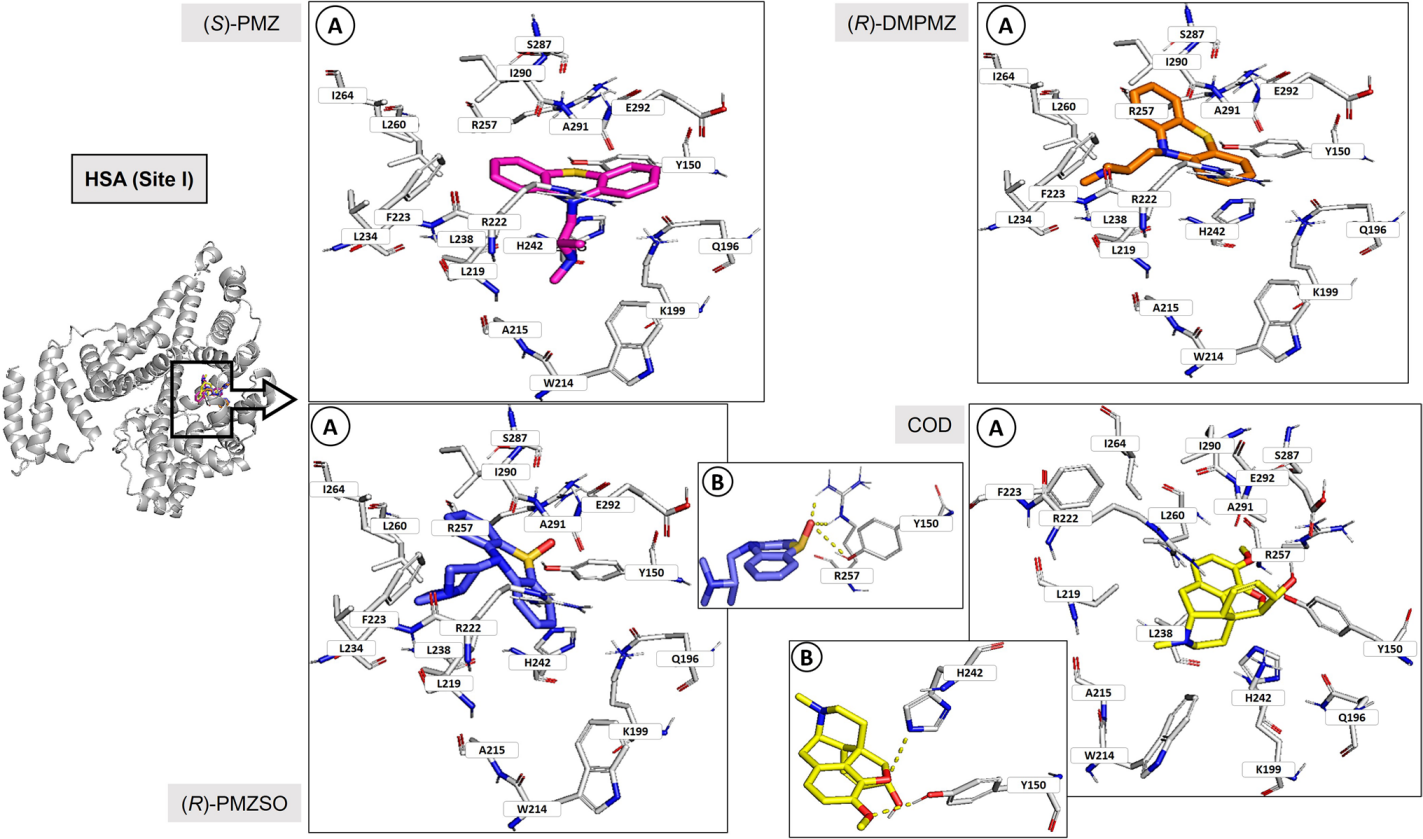
(*S*)-PMZ, (*R*)-DMPMZ, (*R*)-PMZSO, and COD docking with HSA (site I). Carbon, nitrogen, oxygen, and sulphur are represented as white, blue, red, and yellow sticks, respectively. HSA (site I) is represented as grey cartoon and grey sticks, and (*S*)-PMZ, (*R*)-DMPMZ, (*R*)-PMZSO, and COD are represented as magenta, orange, purple, and yellow sticks, respectively. (A) Hydrophobic cleft of the site I; (B) hydrogen bond interactions between (*R*)-PMZSO and Y150 and R257, and COD and H242 and Y150, in site I, represented as yellow dashes. A, alanine; E, glutamic acid; F, phenylalanine; H, histidine; I, isoleucine; K, lysine; L, leucine; Q, glutamine; R, arginine; S, serine; W, tryptophan; Y, tyrosine

Site I is mostly formed by a hydrophobic cleft composed by Tyr150, Phe211, Trp214, Leu219, Ala215, Phe223, Leu234, Leu239, His242, Leu243, Leu260, Ile264, Ile290, Ala291, and three residues, Tyr150, Arg222, and Arg257, that can establish polar contacts. Figure 3 illustrates representative examples of the best binding poses for each HSA:analyte complex at site I. Considering that there was no enantioselectivity and that the binding pose of each enantiomer with HSA is similar, only the interaction of one of the enantiomers with HSA is shown.

PMZ and COD exhibit a very similar binding mode (Fig. 3, (*S*)-PMZ and COD), establishing close hydrophobic contacts with Tyr150, Phe211, Trp214, Ala215, Leu219, Arg222, Phe223, Leu234, Leu238, His242, Leu243, Arg257, Leu260, and Ala291 in the hydrophobic cleft. In addition, both the thiazine of PMZ and the epoxy, hydroxyl, and metoxy groups of COD can establish polar interactions/hydrogen bonds with residue Tyr150. DMPMZ and PMZSO (Fig. 3, (*R*)-DMPMZ and (*R*)-PMZSO), on the other hand, interact more closely with the residues Tyr150, Phe223, Leu234, Leu238, Leu260, Ile264, Ile290, and Ala291 in the hydrophobic cleft. The PMZSO can also establish hydrogen bond interactions with Arg257 through its sulphoxide group.

**Fig. 4:**
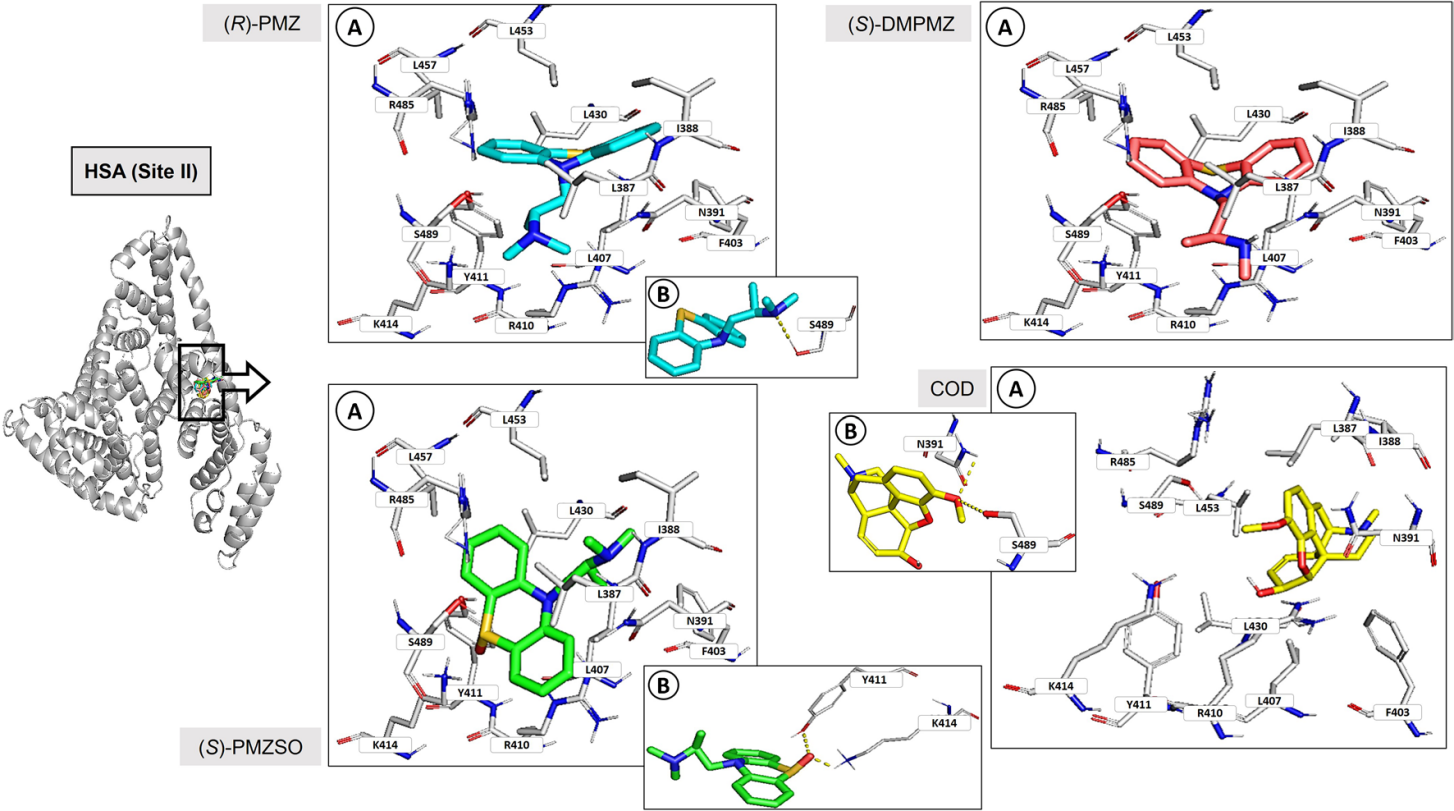
(*R*)-PMZ, (*S*)-DMPMZ, (*S*)-PMZSO, and COD docking with HSA (site II). Carbon, nitrogen, oxygen, and sulphur are represented as white, blue, red, and yellow sticks, respectively. HSA (site II) is represented as grey cartoon and grey sticks, and (*R*)-PMZ, (*S*)-DMPMZ, (*S*)-PMZSO, and COD are represented as cyan, pink, green, and yellow sticks, respectively. A, hydrophobic cleft of site II; B, hydrogen bond interactions between (*R*)-PMZ and S489, (*S*)-PMZSO and Y411 and K414, and COD and N391 and S489, in site II, represented as yellow dashes. F, phenylalanine; K, lysine; L, leucine; N, asparagine; R, arginine; S, serine; Y, tyrosine

Similarly to site I, site II also features a hydrophobic pocket with a cleft formed by residues Leu387, Ile388, Asn391, Phe403, Leu407, Arg410, Tyr411, Lys414, Leu430, Leu453, Leu457, Arg485, and Ser489. As for site I, there was no enantioselectivity and the resulting binding poses of each enantiomer were similar. Hence, only one HSA:analyte complex per enantiomeric pair is represented in Fig. 4.

PMZ and its metabolites occupy the same region within the hydrophobic cleft. In the case of PMZ and DMPMZ, the phenothiazine group establishes hydrophobic interactions with residues Ile388, Leu387, and Leu430, while the propan-amine group establishes hydrophobic contacts with residues Asn391, Leu407, Arg410, Tyr411, and Ser489 (Fig. 4, (*S*)-DMPMZ). Furthermore, PMZ can also form hydrogen bonds with Ser489 (Fig. 4, (*R*)-PMZ). Despite that PMZSO occupies the same region as PMZ and DMPMZ, it exhibits a different binding pose. Consequently, the phenothiazine group instead forms hydrophobic contacts with residues Arg410, Tyr411, Lys414, Arg485, and Ser489, while the propan-amine group engages in hydrophobic contacts with residues Leu387, Ile388, Asn391, Phe403, and Leu453 (Fig. 4, (*S*)-PMZSO). Moreover, the sulphoxide group of PMZSO also establishes hydrogen bonds with residues Tyr411 and Lys414. COD, on the other hand, occupies a distinct region within the hydrophobic cleft, establishing hydrophobic contacts with residues Leu387, Ile388, Asn391, Phe403, Leu407, Arg410, Tyr411, Leu430, Leu453, Arg485, and Ser489 and hydrogen bonds with residues Asn391 and Ser489 (Fig. 4, COD).

Considering the overall interactions established between analytes and HSA, it can be inferred that, both in site I and II, COD, PMZ, and its metabolites engage in hydrophobic interactions with the same residues. Moreover, in site I, for both PMZSO and COD, hydrogen bond interactions occur between the analytes and Tyr150. In site II, hydrogen bond interactions between PMZ and COD with the same residue, Ser489, are also established. Establishing the same type of interactions with the same residues may suggest possible competition between the referred analytes.

To assess whether competitive binding was likely, a final study was carried out to verify if it was possible to bind COD and PMZ at sites I and II simultaneously. The same analysis was carried out for COD in the presence of PMZ metabolites (DMPMZ and PMZSO). To do so, the HSA:ligand complexes (with ligands PMZ, DMPMZ, or PMZSO) from the previous docking calculations were considered as a rigid unit and COD was docked as a flexible ligand, and then the HSA:COD complexes were considered as a rigid unit, and PMZ, DMPMZ, and PMZSO were docked as flexible ligands. The resulting binding free energies are summarized in Tables 4 and S1, in SI.

**Table 4:** Best binding free energies for the docking of COD to the HSA (site I):analyte complex (considering both *R* and *S* enantiomers of PMZ, DMPMZ, and PMZSO) and for the docking of PMZ, DMPMZ, and PMZSO (*R* and *S*) to the HSA (site I):COD complex. Binding free energies are shown in kcal/mol

HSA:analyte receptor	Flexible ligand	Binding free energy (Site I)
HSA:(***R***)-PMZ	COD	− 6.2
HSA:(***S***)-PMZ	COD	− 6.1
HSA:(***R***)-DMPMZ	COD	− 6.0
HSA:(***S***)-DMPMZ	COD	− 6.0
HSA:(***R***)-PMZSO	COD	− 6.2
HSA:(***S***)-PMZSO	COD	− 6.3
HSA:COD	(*R*)-PMZ	− 5.8
HSA:COD	(*S*)-PMZ	− 5.7
HSA:COD	(*R*)-DMPMZ	− 5.7
HSA:COD	(*S*)-DMPMZ	− 5.2
HSA:COD	(*R*)-PMZSO	− 8.3
HSA:COD	(*S*)-PMZSO	− 5.9

Compared with the results obtained for the competitive binding of the analytes towards HSA, the resulting binding free energies were higher (refer to binding free energy in Table 4) when HSA was already binding one analyte, supporting a competitive binding model for the binding of COD at site I. Nevertheless, there is a binding free energy within a 2 kcal/mol difference, which may imply that COD can still bind weaklier in the presence of bound PMZ or metabolites.

The competition between PMZ or its metabolites and COD was more evident for site II. As shown in Table S1, in SI, from the positive (or minimally negative) binding free energies obtained in all situations, and by comparison with the results in Table 3, it is possible to infer that only competition should be possible between PMZ or its metabolites and COD at site II of HSA. Furthermore, visual inspection and analysis of the hydrophobic pocket (Figure S2, SI) indicate that COD and PMZ (or its metabolites) cannot bind simultaneously in that location due to the reduced size of the pocket.

The results thus suggest that the binding of COD and PMZ, or its metabolites, to HSA should be more competitive at site II than site I. Although competitive binding between COD and PMZ should also be more likely at site I, the docking results could not discard the hypothesis that PMZ metabolites (DMPMZ and PMZSO) might bind simultaneously with COD at site I (refer to lower binding free energies for DMPMZ and PMZSO at site II than site I, Table 3, and binding free energies in Table 4).

## Conclusions

In this study, HPAC with an HSA column and docking approach were applied to investigate the interaction between PMZ, its main metabolites, and COD with HSA. The HPAC results demonstrated that PMZ and its metabolite DMPMZ exhibited a high affinity for HSA (> 90% binding), while PMZSO metabolite showed a binding percentage of 80%, and COD displayed a relatively lower affinity (65%). The binding percentages to HSA were also correlated with the lipophilicity of the compounds, as HSA exhibits a strong affinity for lipophilic molecules like PMZ and its metabolites, while COD has lower lipophilicity. PMZ and its metabolites are chiral, but they were not enantioseparated in the investigated elution conditions.

For displacement studies, a zonal chromatography on immobilized HSA column was performed to characterize drug–protein interaction, using warfarin and (*S*)-ibuprofen in the mobile phase as probe compounds, known for binding to sites I and II of HSA, respectively. PMZ revealed more competition with warfarin compared to (*S*)-ibuprofen, but all target compounds exhibited binding to both sites with increasing concentrations of competing compounds in the mobile phase. Docking studies also confirmed the latter experimental results, including the absence of enantioselectivity. In addition, docking results suggested that in site I of HSA, PMZ or its metabolites and COD can bind simultaneously, decreasing its binding affinity. In site II of HSA, high competition was observed, meaning that when one compound is bound, the other attempting to bind cannot.

Extrapolating this situation to what could happen when the soft drink “Purple Drank” is consumed, especially in uncontrolled high doses, a serious problem can be anticipated. Considering that competition between PMZ, and its metabolites, with COD for binding to HSA may occur, an increase of the unbound compound will remain in the bloodstream in its free form, potentially leading to side effects, and/or toxicity, or resulting in overdose. Thus, studying PMZ is crucial not only considering its clinical use but also to address emerging challenges associated with its recreational use. This work is an achievement for the continued research into this drug contributing for promoting public health and ensuring that the therapeutic benefits of PMZ are maximized while potential risks are analysed.

### Supplementary Information

Below is the link to the electronic supplementary material.

Supplementary file1 (DOCX 1077 KB)

## References

[1] Chiappini S, Schifano F, Corkery JM, Guirguis A. Beyond the ‘purple drank’: study of promethazine abuse according to the european medicines agency adverse drug reaction reports. J Psychopharmacol. 2021;35:681–92. 10.1177/026988112095. 33427017 10.1177/026988112095 PMC8278560

[2] Gao S, Zhou X, Lang L, Liu H, Li J, Li H, Wei S, Wang D, Xu Z, Cai H, Zhao Y, Zou W. Simultaneous determination of schisandrin and promethazine with its metabolite in rat plasma by HPLC-MS/MS and its application to a pharmacokinetic study. Int J Anal Chem. 2019;2019:3497045. 10.1155/2019/3497045. 31885590 10.1155/2019/3497045 PMC6925819

[3] Miuli A, Stigliano G, Lalli A, Coladonato M, D’Angelo L, Esposito F, Cappello C, Pettorruso M, Martinotti G, Schifano F, Di Giannantonio M. “Purple drank” (codeine and promethazine cough syrup): a systematic review of a social phenomenon with medical implications. J Psychoact Drugs. 2020;52:453–62. 10.1080/02791072.2020.1797250. 10.1080/02791072.2020.1797250 32748711

[4] Vanapalli SR, Kambhampati SP, Putcha L, Bourne DWA. A liquid chromatographic method for the simultaneous determination of promethazine and three of its metabolites in plasma using electrochemical and uv detectors. J Chromatogr Sci. 2001;39:70–2. 10.1093/chromsci/39.2.70. 11245229 10.1093/chromsci/39.2.70

[5] Coelho MM, Costa I, de Albuquerque ACF, dos Santos Junior FM, Silva B, Silva R, Fernandes C, Remião F, Tiritan ME (2024) Milligram scale enantioresolution of promethazine and its main metabolites, determination of their absolute configuration and assessment of enantioselective effects on human SY-SY5Y cells. J Pharmaceut Biomed. 245. 10.1016/j.jpba.2024.116152. 10.1016/j.jpba.2024.116152 38643704

[6] Agnich LE, Stogner JM, Miller BL, Marcum CD. Purple drank prevalence and characteristics of misusers of codeine cough syrup mixtures. Addict Behav. 2013;38:2445–9. 10.1016/j.addbeh.2013.03.020. 23688907 10.1016/j.addbeh.2013.03.020

[7] Peters RJ Jr, Kelder SH, Markham CM, Yacoubian GS Jr, Peters LA, Ellis A. Beliefs and social norms about codeine and promethazine hydrochloride cough syrup (Cphcs) onset and perceived addiction among urban houstonian adolescents: an addiction trend in the city of lean. J Org Chem. 2003;44:1957–60. 10.2190/NXJ6-U60J-XTY0-09MP. 10.2190/NXJ6-U60J-XTY0-09MP 15237866

[8] Dean L, Kane M. Codeine therapy and CYP2D6 genotype. Europe PMC. 2021. 28520350

[9] Lynch KL, Shapiro BJ, Coffa D, Novak SP, Kral AH. Promethazine use among chronic pain patients. Drug Alcohol Depend. 2015;150:92–7. 10.1016/j.drugalcdep.2015.02.023. 25754939 10.1016/j.drugalcdep.2015.02.023 PMC4389782

[10] Overholser BR, Foster DR. Opioid pharmacokinetic drug-drug interactions. AJMC. 2011;17:276–87. 21999760

[11] Coelho MM, Fernandes C, Remião F, Tiritan ME. Enantioselectivity in drug pharmacokinetics and toxicity: pharmacological relevance and analytical methods. Molecules. 2021;26:1–24. 10.3390/molecules26113113. 10.3390/molecules26113113 PMC8197169 34070985

[12] Rowland M, Matin SB (1973) Kinetics of drug-drug interactions J Pharmacokinet Biopharm. 1. 10.1007/BF01059791.

[13] Bertucci C, Domenici E. Reversible and covalent binding of drugs to human serum albumin: methodological approaches and physiological relevance. Curr Med Chem. 2002;9:1463–81. 10.2174/0929867023369673. 12173977 10.2174/0929867023369673

[14] Harder S, Thurmann P. Clinically important drug interactions with anticoagulants. Clin Pharmacokinet. 1996;30:416–44. 10.2165/00003088-199630060-00002. 8792056 10.2165/00003088-199630060-00002

[15] Roberts JA, Pea F, Lipman J. The clinical relevance of plasma protein binding changes. Clin Pharmacokinet. 2013;52:1–8. 10.1007/s40262-012-0018-5. 23150213 10.1007/s40262-012-0018-5

[16] Bohnert T, Gan LS. Plasma protein binding: from discovery to development. J Pharm Sci. 2013;102(9):2953–94. 10.1002/jps.23614. 23798314 10.1002/jps.23614

[17] Mehvar R. Role of protein binding in pharmacokinetics. Am J Pharm Educ. 2005;69(5).

[18] Bertucci C, Tedesco D. Human serum albumin as chiral selector in enantioselective high-performance liquid chromatography. Curr Med Chem. 2017;24:743–57. 10.2174/0929867324666161118115711. 27855626 10.2174/0929867324666161118115711

[19] Li Z, Hage DS. Analysis of stereoselective drug interactions with serum proteins by high-performance affinity chromatography: a historical perspective. J Pharm Biomed Anal. 2017;144:12–24. 10.1016/j.jpba.2017.01.026. 28094095 10.1016/j.jpba.2017.01.026 PMC5505820

[20] Cardoso T, Almeida AS, Remião F, Fernandes C (2021) Enantioresolution and binding affinity studies on human serum albumin: recent applications and trends. Chemosensors. 9. 10.3390/chemosensors9110304.

[21] Siddiqui S, Ameen F, Rehman S, Sarwar T, Tabish M (2021) Studying the interaction of drug/ligand with serum albumin. J Mol Liq. 336. 10.1016/j.molliq.2021.116200.

[22] Wanat K, Brzezińska E, Sobańska AW. Aspects of drug-protein binding and methods of analyzing the phenomenon. Curr Pharm Des. 2018;24:2974–85. 10.2174/1381612824666180808145320. 30088445 10.2174/1381612824666180808145320

[23] Rodriguez EL, Poddar S, Iftekhar S, Suh K, Woolfork AG, Ovbude S, Pekarek A, Walters M, Lott S, Hage DS (2020) Affinity chromatography: a review of trends and developments over the past 50 years. J Chromatogr B Biomed Appl. 1157. 10.1016/j.jchromb.2020.122332. 10.1016/j.jchromb.2020.122332 PMC7584770 32871378

[24] Almeida AS, Cardoso T, Cravo S, Tiritan ME, Remião F, Fernandes C. Binding studies of synthetic cathinones to human serum albumin by high-performance affinity chromatography. J Chromatogr B Biomed Appl. 2023. 10.1016/j.jchromb.2023.123836. 10.1016/j.jchromb.2023.123836 37494753

[25] Ovbude ST, Tao P, Li Z, Hage DS (2022) Characterization of binding by repaglinide and nateglinide with glycated human serum albumin using high-performance affinity microcolumns. J Sep Sci. 45(23). 10.1002/jssc.202200686. 10.1002/jssc.202200686 PMC10012256 36168862

[26] Andrisano V, Gotti R, Recanatini M, Cavalli A, Varoli L, Bertucci C. Stereoselective binding of 2-(4-biphenylyl)-3-substituted-3-hydroxypropionic acids on an immobilised human serum albumin chiral stationary phase. J Chromatogr B Biomed Appl. 2002;768:137–45. 10.1016/S0378-4347(01)00493-5. 10.1016/S0378-4347(01)00493-5 11939547

[27] Barbato F, Carpentiero C, Grumetto L, Rotonda MI. Enantioselective retention of beta-blocking agents on human serum albumin and alpha 1-acid glycoprotein HPLC columns: relationships with different scales of lipophilicity. Eur J Pharm Sci. 2009;38(5):472–8. 10.1016/j.ejps.2009.09.011. 19778607 10.1016/j.ejps.2009.09.011

[28] do Carmo JP, Phyo YZ, Palmeira A, Tiritan ME, Afonso C, Kijjoa A, Pinto MMM, Fernandes C. Enantioseparation, recognition mechanisms and binding of xanthones on human serum albumin by liquid chromatography. Bioanalysis. 2019;11:1255–74. 10.4155/bio-2019-0074. 31298568 10.4155/bio-2019-0074

[29] Lambrinidis G, Vallianatou T, Tsantili-Kakoulidou A. In vitro, in silico and integrated strategies for the estimation of plasma protein binding. A review Adv Drug Deliv Rev. 2015;23(86):27–45. 10.1016/j.addr.2015.03.011. 10.1016/j.addr.2015.03.011 25819487

[30] Meng X, Zhang H, Mezei M. Molecular docking: a powerful approach for structure-based drug discovery. Curr Comput-Aided Drug Des. 2011;7:146–57. 10.2174/157340911795677602. 21534921 10.2174/157340911795677602 PMC3151162

[31] He L, Wang Z, Wang Y, Liu X, Yang Y, Gao Y, Wang X, Liu B, Wang X (2016) Studies on the interaction between promethazine and human serum albumin in the presence of flavonoids by spectroscopic and molecular modeling techniques. Colloids Surf B: Biointerfaces. 10.1016/j.colsurfb.2016.06.001. 10.1016/j.colsurfb.2016.06.001 27315330

[32] Judis J. Binding of codeine, morphine, and methadone to human serum proteins. J Pharm Sci. 1977;66:802–5. 874779 10.1002/jps.2600660615

[33] Judis J. Protein concentration effects on binding of 14c-codeine, i4c-morphine, and 3h-methadone to human serum albumin. J Pharm Sci. 1980;69:71–3. 10.1002/jps.2600690119. 7354447 10.1002/jps.2600690119

[34] Sudlow G, Birkett DJ, Wade DN. The characterization of two specific drug binding sites on human serum albumin. Mol Pharmacol. 1975;11:824–32. 1207674

[35] Hage DS. High-performance affinity chromatography: a powerful tool for studying serum protein binding. J Chromatogr B. 2002;768:3–30. 10.1016/S0378-4347(01)00482-0 11939555

[36] Hage DS, Anguizola J, Barnaby O, Jackson A, Yoo MJ, Papastavros E, Pfaunmiller E, Sobansky M, Tong Z. Characterization of drug interactions with serum proteins by using high-performance affinity chromatography. Curr Drug Metab. 2011;12:313–28. 10.2174/138920011795202938. 21395530 10.2174/138920011795202938 PMC3174051

[37] Pistolozzi M, Fortugno C, Franchini C, Corbo F, Muraglia M, Roy M, Félix G, Bertucci C. Species-dependent binding of tocainide analogues to albumin: Affinity chromatography and circular dichroism study. J Chromatogr B Biomed Appl. 2014;968:69–78. 10.1016/j.jchromb.2014.01.007. 10.1016/j.jchromb.2014.01.007 24472243

[38] Kim HS, Wainer IW. Rapid analysis of the interactions between drugs and human serum albumin (HSA) using high-performance affinity chromatography (HPAC). J Chromatogr B Analyt Technol Biomed Life Sci. 2008;870:22–6. 10.1016/j.jchromb.2008.05.029. 18554995 10.1016/j.jchromb.2008.05.029 PMC2556154

[39] Rose PW, Prlić A, Bi C, Bluhm WF, Christie CH, Dutta S, Green RK, Goodsell DS, Westbrook JD, Woo J, Young J, Zardecki C, Berman HM, Bourne PE, Burley SK (2015) The RCSB Protein Data Bank: views of structural biology for basic and applied research and education. Nucleic Acids Res. 43(D345-D356). 10.1093/nar/gku1214. 10.1093/nar/gku1214 PMC4383988 25428375

[40] Hutchison GR, Morley C, James C, Swain C, De Winter H, Vandermeersch T, O’Boyle NM. Open Babel Documentation. 2011;151. 10.1186/1758-2946-3-33 PMC3198950 21982300

[41] Dennington RD, Keith TA, Millam JM. GaussView 5.0. Wallingford, US. 2008.

[42] Trott O, Olson AJ. AutoDock Vina: improving the speed and accuracy of docking with a new scoring function, efficient optimization and multithreading. J Comput Chem. 2010;31:455–61. 10.1002/jcc.21334. 19499576 10.1002/jcc.21334 PMC3041641

[43] Schrödinger LL. O sistema gráfico molecular PyMOL. 1–8 edn. 2015.

[44] Daina A, Michielin O, Zoete V (2017) SwissADME: a free web tool to evaluate pharmacokinetics, druglikeness and medicinal chemistry friendliness of small molecules. Sci Rep. 7. 10.1038/srep42717. 10.1038/srep42717 PMC5335600 28256516

[45] Kim HS, Kye YS, Hage DS. Development and evaluation of N-hydroxysuccinimide-activated silica for immobilizing human serum albumin in liquid chromatography columns. J Chromatogr A. 2004;1049:51–61. 10.1016/j.chroma.2004.08.010. 15499917 10.1016/j.chroma.2004.08.010

[46] Bertucci C, Cimitan S, Riva A, Morazzi P. Binding studies of taxanes to human serum albumin by bioaffinity chromatography and circular dichroism. J Pharm Biomed Anal. 2006;42:81–7. 10.1016/j.jpba.2005.12.002. 16413734 10.1016/j.jpba.2005.12.002

[47] Haginaka J. Recent progresses in protein-based chiral stationary phases for enantioseparations in liquid chromatography. J Chromatogr B Analyt Technol Biomed Life Sci. 2008;875(1):12–9. 10.1016/j.jchromb.2008.05.022. 18515198 10.1016/j.jchromb.2008.05.022

[48] Chen H, Gong Z, Zhang Z. Coupling microdialysis with flow-injection chemiluminescence detection for a protein–drug interaction study. J Pharm Biomed Anal. 2006;41:1412–7. 10.1016/j.jpba.2006.02.050. 16616825 10.1016/j.jpba.2006.02.050

[49] Ghuman J, Zunszain PA, Petitpas I, Bhattacharya AA, Otagiri M, Curry SH. Structural basis of the drug-binding specificity of human serum albumin. J Mol Biol. 2005;353:38–52. 10.1016/j.jmb.2005.07.075. 16169013 10.1016/j.jmb.2005.07.075

[50] Lázaro E, Lowe PJ, Birand X, Faller B. New approach to measure protein binding based on a parallel artificial membrane assay and human serum albumin. J Med Chem. 2008;51:2009–17. 10.1021/jm7012826. 18348514 10.1021/jm7012826

[51] More J, Bulmer M (2013) Human serum albumin: a multifunctional plasma protein. In: Wiley (ed) Production of Plasma Proteins for Therapeutic Use. pp 159–183. 10.1002/9781118356807.ch12.

[52] Völgyi G, Marosi A, Takács-Novák K, Avdeef A. Salt solubility products of diprenorphine hydrochloride, codeine and lidocaine hydrochlorides and phosphates – novel method of data analysis not dependent on explicit solubility equations. ADMET & DMPK. 2013;1:48–62. 10.5599/admet.1.4.24. 10.5599/admet.1.4.24

[53] EH-Haj BM. Metabolic N-dealkylation and N-oxidation as elucidators of the role of alkylamino moieties in drugs acting at various receptors. Molecules. 2021;26:1917. 10.3390/molecules26071917. 33805491 10.3390/molecules26071917 PMC8036657

[54] Nakamura K, Yokoi T, Inoue K, Shimada N, Ohashi N, Kume T, Kamataki T. CYP2D6 is the principal cytochrome P450 responsible for metabolism of the histamine H1 antagonist promethazine in human liver microsomes. Pharmacogenetics. 1996;16:449–57. 10.1097/00008571-199610000-00009. 10.1097/00008571-199610000-00009 8946477

[55] Taylor G, Houston JB, Shaffer J, Maweri G. Pharmacokinetics of promethazine and its sulphoxide metabolite after intravenous and oral administration to man. Br J Clin Pharmacol. 1983;15:287–93. 10.1111/j.1365-2125.1983.tb01501.x. 6849764 10.1111/j.1365-2125.1983.tb01501.x PMC1427776

[56] Zhao X, Li Q, Chen J, Xiao C, Bian L, Zheng J, Zheng X, Li Z, Zhang Y (2014) Exploring drug–protein interactions using the relationship between injection volume and capacity factor. J Chromatogr A. 137–144. 10.1016/j.chroma.2014.03.017. 10.1016/j.chroma.2014.03.017 24666938

[57] He L, Wang X, Liu B, Wang J, Sun Y, Gao E, Xu S. Study on the interaction between promethazine hydrochloride and bovine serum albumin by fluorescence spectroscopy. J Lumin. 2011;131(2):285–90. 10.1016/j.jlumin.2010.10.014. 10.1016/j.jlumin.2010.10.014

[58] Noctor TAG, Diaz-Perez MJ, Wainer IW. Use of a human serum albumin-based stationary phase for high-performance liquid chromatography as a tool for the rapid determination of drug-plasma protein binding. J Pharm Sci. 1993;82(6):675–6. 10.1002/jps.2600820629. 8331550 10.1002/jps.2600820629

[59] Martínez-Gómez MA, Villanueva-Camañas RM, Sagrado S, Medina-Hernández MJ. Evaluation of enantioselective binding of basic drugs to plasma by ACE. J Electrophor. 2007;28:3056–63. 10.1002/elps.200700222. 10.1002/elps.200700222 17661317

[60] Zhang XX, Hong F, Chang L. Enantiomeric separation of promethazine and D, L-a-amino-b-[4-(1,2dihydro-2-oxo-quinoline)] propionic acid drugs by capillary zone electrophoresis using albumin as chiral selectors. Anal Chim Acta. 1999;392:175–81. 10.1016/S0003-2670(99)00248-2. 10.1016/S0003-2670(99)00248-2

[61] Deb PK, Al-Attraqchi O, Prasad MR, Tekade RK. Protein and tissue binding: implication on pharmacokinetic parameters. In: Tekade RK, editor. Dosage form design considerations, vol. i. Advances in Pharmaceutical Product Development and Research: Academic Press; 2018. p. 371–99.

[62] Sharma AS, Anandakumar S, Ilanchelian M. In vitro investigation of domain specific interactions of phenothiazine dye with serum proteins by spectroscopic and molecular docking approaches. RSC Adv. 2014;4:36267–81. 10.1039/c4ra04630g. 10.1039/c4ra04630g

[63] Pérez DI, Pistolozzi M, Palomo V, Redondo M, Fortugno C, Gil C, Felix G, Martinez A, Bertucci C. 5-Imino-1,2–4-thiadiazoles and quinazolines derivatives as glycogen synthase kinase 3b (GSK-3b) and phosphodiesterase 7 (PDE7) inhibitors: Determination of blood–brain barrier penetration and binding to human serum albumin. Eur J Pharm Sci. 2012;45:677–84. 10.1016/j.ejps.2012.01.007. 22306656 10.1016/j.ejps.2012.01.007

[64] Bai G, Pan Y, Zhang Y, Li Y, Wang J, Wang Y, Teng W, Jin G, Geng F, Cao J (2023) Research advances of molecular docking and molecular dynamic simulation in recognizing interaction between muscle proteins and exogenous additives. Food Chem. 429. 10.1016/j.foodchem.2023.136836. 10.1016/j.foodchem.2023.136836 37453331

[65] Patil SM, Maruthi KR, Bajpe SN, Vyshali VM, Sushmitha S, Akhila C, Ramu R. Comparative molecular docking and simulation analysis of molnupiravir and remdesivir with SARS-CoV-2 RNA dependent RNA polymerase (RdRp). Bioinformation. 2021;17:932–9. 10.6026/97320630017932. 35655903 10.6026/97320630017932 PMC9148593

[66] Mohan V, Gibbs AC, Cummings MD, Jaeger EP, DesJarlais RL. Docking: successes and challenges. Curr Pharm Des. 2005;11:323–33. 10.2174/1381612053382106. 15723628 10.2174/1381612053382106

[67] Kontoyianni M, McClellan LM, Sokol GS. Evaluation of docking performance: comparative data on docking algorithms. J Med Chem. 2004;47:558–65. 10.1021/jm0302997. 14736237 10.1021/jm0302997

