# Supplementary material for: Binding studies of promethazine and its metabolites with human serum albumin by high-performance affinity chromatography and molecular docking in the presence of codeine
Source: Anal Bioanal Chem. 2024 Jul 4;416(20):4605–18. doi: 10.1007/s00216-024-05409-3 (PMC11294390; doi:10.1007/s00216-024-05409-3)
Supplement: Supplementary file 1 — Supplementary file1 (DOCX 1077 KB) [file 216_2024_5409_MOESM1_ESM.docx]

**Binding studies of promethazine and its metabolites with human serum albumin by high-performance affinity chromatography and molecular docking in the presence of codeine**

Maria Miguel Coelho^1,2,3^, Rita Lima^1,2^, Ana Sofia Almeida^1,2,3^, Pedro Alexandrino Fernandes^4^, Fernando Remião^3^, Carla Fernandes^1,2*^, and Maria Elizabeth Tiritan^1,2,5*^

^1^ Laboratory of Organic Chemistry and Pharmaceuticals, Department of Chemical Sciences, Faculty of Pharmacy of the University of Porto, 4050-313 Porto, Portugal

^2^ CIIMAR-Interdisciplinary Center for Marine and Environmental Research University of Porto, Porto de Leixões Cruise Terminal, 4450-208, Matosinhos, Portugal

^3^ UCIBIO-Applied Molecular Biosciences Unit, Laboratory of Toxicology, Department of Biological Sciences, Faculty of Pharmacy, University of Porto, 4050-313, Porto, Portugal

^4^ LAQV, REQUIMTE, Departamento de Química e Bioquímica, Faculdade de Ciências Universidade do Porto, Rua do Campo Alegre, s/n, 4169-007 Porto, Portugal

^5^ 1H-TOXRUN – One Health Toxicology Research Unit, University Institute of Health Sciences (IUCS), CESPU, CRL, 4585-116 Gandra, Portugal

* Correspondence author: beth@ff.up.pt; cfernandes@ff-up.pt

**3. Results and discussion**

3.2 Displacement studies


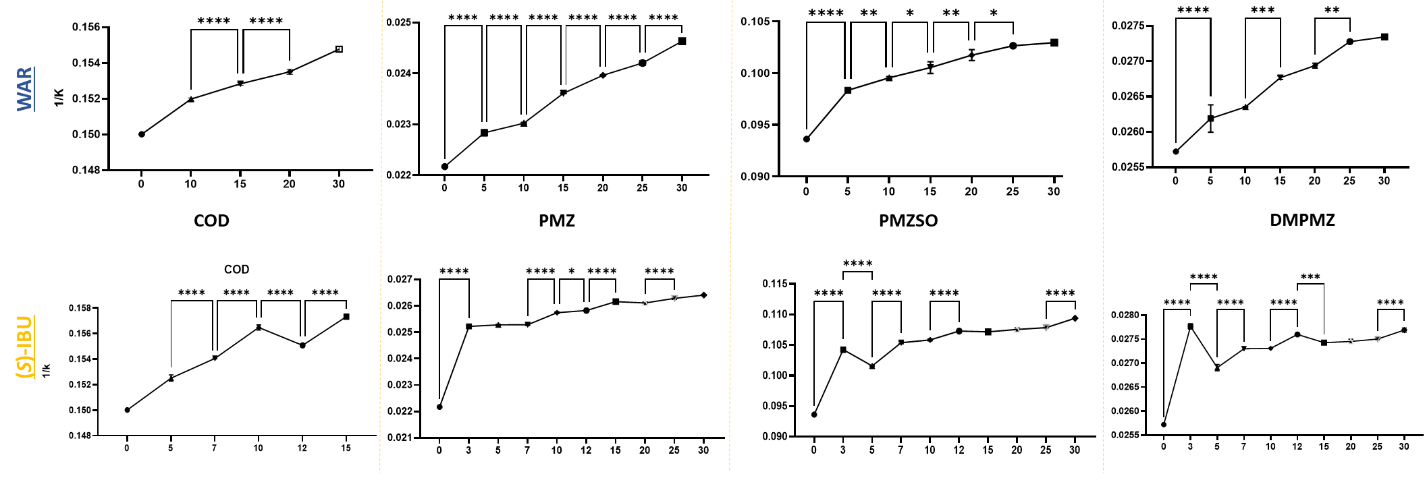


**Figure S1.** Displacement chromatography experiments with PMZ and metabolites and the COD in the absence and presence of increasing concentrations of the competitors, warfarin (War) and (S)-Ibuprofen ((S)-IBU), expressed statistically by the graph of 1/k of the analyte versus the concentrations of the competing compounds. **** extremely significant, *** extremely significant, ** very significant, * significant

3.3 Docking studies

**Table S1.** Binding free energies for the top conformation of COD towards the HSA (site II): analyte complex (considering both R and S enantiomers of PMZ, DMPMZ and PMZSO) and for the top conformation of PMZ, DMPMZ and PMZSO (R and S) towards the HSA (site II): COD complex. Binding free energies are shown in kcal/mol.

| HSA:analyte receptor | Flexible ligand | Binding free energy (Site II) |
| --- | --- | --- |
| HSA:(*R*)-PMZ | COD | 11.3 |
| HSA:(*S*)-PMZ | COD | 8.8 |
| HSA:(*R*)-DMPMZ | COD | 28.9 |
| HSA:(*S*)-DMPMZ | COD | 12.3 |
| HSA:(*R*)-PMZSO | COD | 11.5 |
| HSA:(*S*)-PMZSO | COD | 11.5 |
| HSA:COD | (*R*)-PMZ | 4.2 |
| HSA:COD | (*S*)-PMZ | 3.5 |
| HSA:COD | (*R*)-DMPMZ | -1.9 |
| HSA:COD | (*S*)-DMPMZ | 0.7 |
| HSA:COD | (*R*)-PMZSO | -0.3 |
| HSA:COD | (*S*)-PMZSO | 0.8 |


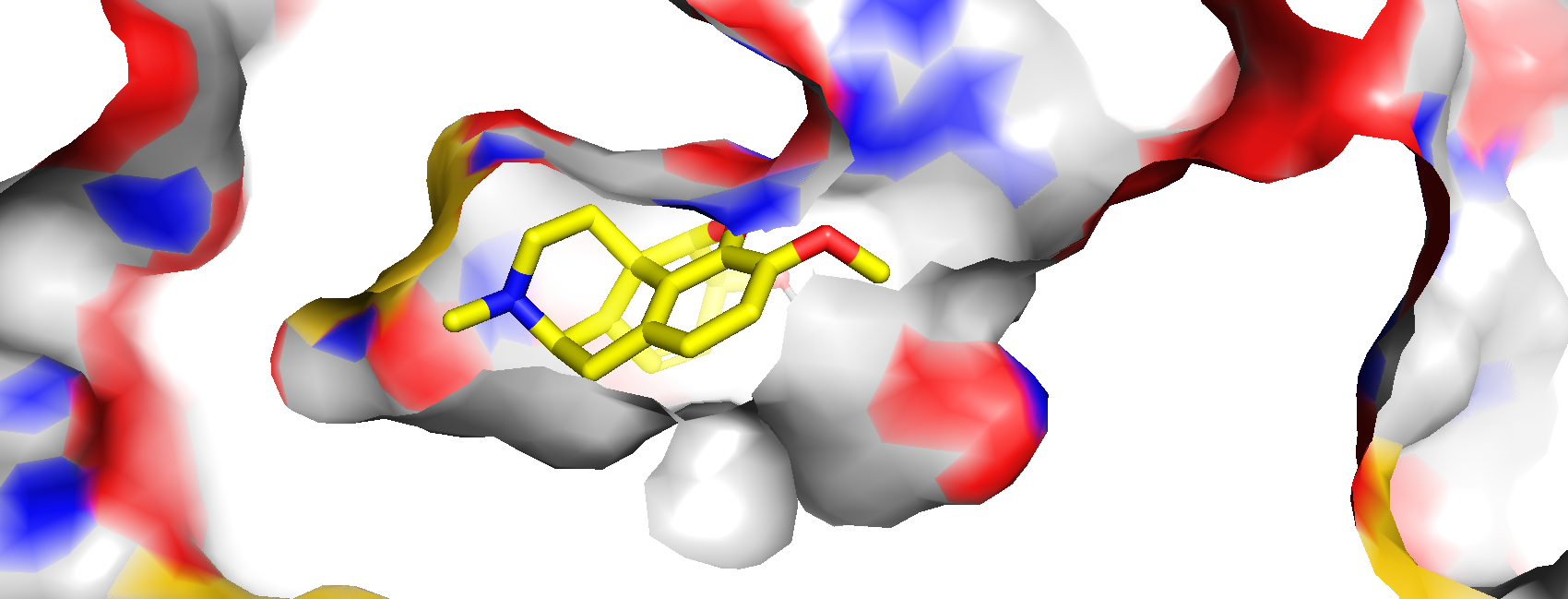


**Figure S2.** Representation of the hydrophobic pocket with COD in site II of the HSA. COD is represented as yellow sticks and HSA is represented as grey surface.
